# Brain Interleukin-1β and the Intrinsic Receptor Antagonist Control Peripheral Toll-Like Receptor 3-Mediated Suppression of Spontaneous Activity in Rats

**DOI:** 10.1371/journal.pone.0090950

**Published:** 2014-03-12

**Authors:** Masanori Yamato, Yasuhisa Tamura, Asami Eguchi, Satoshi Kume, Yukiharu Miyashige, Masayuki Nakano, Yasuyoshi Watanabe, Yosky Kataoka

**Affiliations:** 1 Cellular Function Imaging Team, RIKEN Center for Life Science Technologies, Kobe, Japan; 2 Pathophysiological and Health Science Team, RIKEN Center for Life Science Technologies, Kobe, Japan; 3 Department of Physiology, Osaka City University Graduate School of Medicine, Osaka, Japan; Temple University School of Medicine, United States of America

## Abstract

During acute viral infections such as influenza, humans often experience not only transient fever, but also prolonged fatigue or depressive feelings with a decrease in social activity for days or weeks. These feelings are thought to be due to neuroinflammation in the brain. Recent studies have suggested that chronic neuroinflammation is a precipitating event of various neurological disorders, but the mechanism determining the duration of neuroinflammation has not been elucidated. In this study, neuroinflammation was induced by intraperitoneal injection of polyriboinosinic:polyribocytidylic acid (poly I:C), a Toll-like receptor-3 agonist that mimics viral infection in male Sprague-Dawley rats, and then investigated how the neuroinflammation shift from acute to the chronic state. The rats showed transient fever and prolonged suppression of spontaneous activity for several days following poly I:C injection. NS-398, a cyclooxygenase-2 inhibitor, completely prevented fever, but did not improve spontaneous activity, indicating that suppression of spontaneous activity was not induced by the arachidonate cascade that generated the fever. The animals overexpressed interleukin (IL)-1β and IL-1 receptor antagonist (IL-1ra) in the brain including the cerebral cortex. Blocking the IL-1 receptor in the brain by intracerebroventricular (i.c.v.) infusion of recombinant IL-1ra completely blocked the poly I:C-induced suppression of spontaneous activity and attenuated amplification of brain interferon (IFN)-α expression, which has been reported to produce fatigue-like behavior by suppressing the serotonergic system. Furthermore, i.c.v. infusion of neutralizing antibody for IL-1ra prolonged recovery from suppression of spontaneous activity. Our findings indicated that IL-1β is the key trigger of neuroinflammation and that IL-1ra prevents the neuroinflammation entering the chronic state.

## Introduction

When humans are infected with a virus such as influenza, acute inflammation occurs, and pro-inflammatory cytokines including interleukin (IL)-1β and/or antiviral cytokines including interferons (IFNs) are produced in the periphery. Even during a peripheral infection, humans often experience not only fever, but also abnormal psychological and somatic feelings, which are called depressive symptoms. These symptoms consist of early-onset anorexia and fatigue sensation as well as late-onset cognitive impairment, etc. [1]–[3], suggesting that peripherally produced cytokines affect brain function. Cytokine expression in the brain is also thought to be involved in depressive symptoms. Recent studies have revealed that this neuroimmunological response, called neuroinflammation, is an important precipitating event of various neurological disorders [4], [5]. These observations suggest that balancing induction and suppression of neuroinflammation is important for maintaining healthy brain function.

Intraperitoneal (i.p.) injection of polyriboinosinic:polyribocytidylic acid (poly I:C), a synthetic double-stranded RNA, mimics viral infection. Injected poly I:C is recognized by Toll-like receptor (TLR) 3, which is a specific receptor for the double-stranded RNA structure. In the periphery, TLR3 is expressed in macrophages, dendritic cells, and intestinal epithelial cells. TLR3 uses Toll/IL-1R domain-containing adaptor molecule (TICAM)-1 to activate downstream interferon regulatory factor (IRF)-3 and NF-κB, and TLR3 induces production of anti-viral type I IFNs and inflammatory cytokines such as IL-1β, IL-6, and tumor necrosis factor (TNF)-α [6]. Katafuchi et al. [7] reported that peripheral injection of poly I:C in a single dose suppresses voluntary running wheel activity for more than a week following transient fever in rats. Such a behavioral suppression was accompanied by prolonged upregulation of IFN-α mRNA in the cerebral cortex, hippocampus, and hypothalamic regions for more than a week after the injection. IFN-α in the brain has been demonstrated to modulate serotonergic systems by up-regulating serotonin transporter (5-HTT) [8], [9]. The study suggests that peripheral injection of poly I:C even in a single dose induces neuroinflammation in the brain persisting for a long time (at least several days). Thus, the animal model will be useful to study the mechanism of exacerbation and suppression of neuroinflammation.

Other inflammatory cytokines than IFN-α have been also reported to play an important role in the expression of sickness- and depressive symptoms [10]. IL-1β is implicated in such symptoms and also in chronic neurodegenerative processes involved in vascular dementia and Alzheimer's disease [11], [12]. Activation of IL-1 receptor type 1 (IL-1R1) by IL-1β leads to NF-κB activation, which, in turn, increases inflammation [13], [14]. IL-1 receptor antagonist (IL-1ra), a member of the IL-1 family, is the endogenous competitive antagonist for IL-1 receptors and counteracts the pro-inflammatory action of IL-1β [15]. Some reports have suggested that endogenous IL-1ra has neuroprotective effects during brain inflammation [15], [16]. Therefore, the functional balance between IL-1β and IL-1ra may maintain the function of IL-1 signaling.

In the present study, we identified the roles of IL-1β and IL-1ra in the brain, focusing on the mechanism of exacerbation and suppression of neuroinflammation which is induced by peripheral injection of poly I:C, and on the shift of neuroinflammation from acute state to chronic state that is the persistent condition of neuroinflammation even after disappearance of peripheral inflammation. We also studied the functional relationship between IL-1β and IFN-α in the brain.

## Materials and Methods

### Animals

Male Sprague–Dawley rats (Shizuoka Laboratory Animal Cooperative; 8 weeks old, Shizuoka, Japan) were used in this study. The animals were housed in a cage with a raised mesh base under constant environmental conditions (room temperature, 22–23°C; relative humidity, 50%–60%) and a 12-h light-dark cycle (08:00/20:00). Food and water were provided *ad libitum*. All experimental protocols were approved by the Ethics Committee on Animal Care and Use of the RIKEN Center for Life Science Technologies (MAH19-01-13), and were performed in accordance with the Principles of Laboratory Animal Care (NIH publication No. 85–23, revised 1985). All efforts were made to minimize animal suffering and the number of animals used for the studies.

### Induction of depressive behavior

Depressive behavior was induced by i.p. injection of poly I:C (Amersham Pharmacia Biotech, Piscataway, NJ) dissolved in saline at a dose of 3 mg/kg. The injection was performed between 10:00 and 11:00. NS-398 (a selective Cyclooxygenase-2 (COX-2) inhibitor, Sigma; 4 mg/kg body weight) or vehicle (500 µl 50% dimethyl sulfoxide [DMSO] in saline) was administered i.p. 5 min before and 4 h after poly I:C injection. Rat recombinant (rr) IL-1β (PEPROTECH, Rocky Hill, NJ) dissolved in 0.1 M phosphate-buffered saline (PBS) was administered intraperitoneally at a dose of 30 µg/kg body weight.

### Measurement of spontaneous activity

Spontaneous activity was measured during the dark period (12 h) when rats were active in their home cages. Activity was quantitated with an infrared beam sensor (NS-AS01; Neuroscience Inc., Tokyo, Japan) placed about 15 cm above the center of the cage and analyzed using Clock Lab (Neuroscience Inc.). As the rat moves within the cage, the invisible infrared beams are disrupted, and the machine records the number of broken beams and generates an ambulatory count as a measure of spontaneous activity. Rats were placed in the cage for 7 days to allow for habituation to the environment. The count during the dark period was considered the spontaneous activity. The mean counts of last three days in habituation period were used as baseline activity. After that, animals received an intracerebroventricular (i.c.v.) infusion or i.p. injection. The percent change in spontaneous activity was observed. We tested the spontaneous activity of isolated rats because this monitoring system cannot count spontaneous activity of multiple animals in the same cage concurrently.

### Measurement of rectal temperature

Variation in rectal temperature was recorded using a telethermometer (Yellow Springs Instrument Co., Inc., Yellow Springs, OH) by inserting the thermister probe to a depth of 2.5 cm into the rectum of the rat. The animals were lightly restrained by holding them in the hand, and the steady readout was achieved within 15 sec after probe insertion. The rectal temperature of each animal was recorded just before drug administration and at 1, 3, 5, 7, and 24 h after poly I:C injection.

### Surgical procedure for i.c.v. infusion using a microinfusion pump

Rats were subcutaneously implanted with a microinfusion pump “iPRECIO” (Model: SMP-200, Primetech Corporation, Tokyo, Japan) using the modified method of Abe et al. [17]. Briefly, 7 days before the start of infusion, a chronic i.c.v. cannula (Brain Infusion Kit 1; Alzet, Cupertino, CA) was inserted into the left lateral ventricle of the brain under isoflurane anesthesia. The cannula was stereotaxically positioned in the lateral ventricle according to the brain atlas of Paxinos and Watson [18] at the following coordinates from bregma: AP, -0.8 mm; ML, 1.5 mm; DV, -3.2 mm. The cannula was secured to the skull of the animal with stainless steel screws and dental cement. A main body of the iPRECIO was implanted subcutaneously under the back of the neck and the catheter of iPRECIO was connected to the cannula. After closing the incision, penicillin G potassium (6000 U/day) was injected intramuscularly. All rats were maintained in their home cages to recover from the surgery until the experiment.

The infusion start time, infusion end time, and infusion rate were programmed with the iPRECIO Management Software Ver. 1.3 (Primetech Corporation). Infusion rate was set at the rate of 1.0 µl/h in all i.c.v. infusion procedures. The 0.1 M PBS was infused during the recovery for a week after surgery, and the solution in the iPRECIO was changed from 0.1 M PBS to following substances using 26G needle: rrIL-1β (PEPROTECH; dissolved in 0.1 M PBS) at a dose of 30 ng/day for 24 h or 30 ng/day for 16 h; rrIL-1ra (R&D Systems, Inc.; dissolved in 0.1 M PBS containing 0.1% bovine serum albumin) at a dose of 1 µg/day or vehicle for 5 days; neutralizing antibody for IL-1ra (R&D Systems, Inc.; diluted in 0.1 M PBS) at a dose of 2400 ng/day or vehicle for 14 days. Procedures for changing substances in the reservoir were conducted under light anesthesia using isoflurane inhalation applied through a face mask.

### RT-PCR analysis

After sacrificing the rats under deep anesthesia, brains were quickly removed and placed on ice. The cerebral cortex, hippocampus, hypothalamus, and cerebellum were dissected, and the samples were immediately frozen in liquid nitrogen, stored at −80°C, and then subjected to mRNA assay. Total RNA was isolated from brain tissue using an ISOGEN kit (Nippon Gene, Tokyo, Japan) according to the manufacturer's protocol. After precipitation, the RNA was resuspended in RNase-free water, and the concentration was measured by absorbance at a wavelength of 260 nm. One microgram of RNA was used for reverse transcription (RT). RT and polymerase chain reaction (PCR) assays were performed using a Mastercycler ep (Eppendorf, Tokyo, Japan) and Ready-To-Go PCR Beads (GE Healthcare, Little Chalfont, Buckinghamshire, UK). To monitor the efficiency of cDNA synthesis, glyceraldehyde-3-phosphate dehydrogenase (GAPDH) was amplified as an internal control. Thirty-two PCR cycles were used, and were shown to not cause saturation of transcripts. The gene accession numbers and primer sequences for amplification of cDNA are as follows: IL-1β (**NM_031512**) sense 5′-CAAAAATGCCTCGTGCTGTC-3′, antisense 5′-CCGACCATTGCTGTTTCCTA-3′; IL-1ra (**NM_022194**) sense 5′-TCTGCAGGGGACCTTACAGT-3′, antisense 5′-GGTCTTCCTGGAAGTAGAAC-3′; IFN-α (**NM_001271218**) sense 5′-CCTGCCTCACACTCATAACC-3′, antisense 5′-CTTCTCTCAGTCTTCCCATC-3′; GAPDH (**NM_017008**) sense 5′-AAAAGGGTCATCATCTCCGC-3′, antisense 5′-CAGCATCAAAGGTGGAGGAA-3′. The PCR reaction was started with denaturation at 94°C for 5 min, followed by 32 cycles of 94°C for 30 s, 55°C for 30 s, and 72°C for 1 min, with a final extension at 72°C for 10 min. Negative technical control without cDNA was used for each set of reactions. The predicted sizes of PCR products are 378 bp for IL-1β, 525 bp for IL-1ra, 488 bp for IFN-α, and 544 bp for GAPDH. The PCR products were electrophoresed in 2.0% agarose gels containing ethidium bromide in 1× Tris-acetate-EDTA buffer. Photographs were taken and analyzed with a densitometer DMU-33C densitometer (Advantec Toyo, Tokyo, Japan). In this study, real-time RT-PCR study was also performed. The cDNA was synthesized from 1 µg of total RNA using reverse transcriptase with gDNA elaser (Takara Bio, Shiga, Japan). Complementary DNA (10 ng) was amplified by real time PCR. Real time PCR was performed in the Thermal Cycler Dice Real Time System (Takara Bio), using the standard protocols with the KAPA SYBR qPCR Master Mix (Kapa Biosystems, Boston, MA). The relative expressions of IL-1β and IFN-α mRNA were calculated after normalization to GAPDH mRNA. The gene accession numbers and primer sequences for amplification of cDNA are as follows: rat IL-1β (**NM_031512**) sense 5′-TTGTCGAGATGCTGCTGTGAG-3′, antisense 5′-TCCTTGTGCAAGTGTCTGAAGC-3′; rat IFN-α (**NM_001271218**) sense 5′-TCATTCTGTAATGACCTCCAGCA-3′, antisense 5′-CTCTCCAGACTTCTGCTCTGACC-3′; rat GAPDH (**NM_017008**) sense 5′-GGCTGGGGCTCACCTGAAG-3′, antisense 5′-GCCCTTCCACGATGCCAAAG-3′.

### Immunoblotting

For the in vivo i.c.v. infusion study, we verified that the anti-mouse IL-1ra neutralizing antibody recognizes rrIL-1ra. rrIL-1ra (300 and 1000 pg; R&D Systems, Inc.) was separated with SDS-PAGE (12.5% acrylamide gel; e-PAGEL, ATTO Corporation, Tokyo, Japan) and transferred onto a PVDF membrane (0.45 µm pore size; Millipore, Billerica, MA) at 15 V for 45 min in transfer buffer. The membrane was preblocked with 5.0% skim milk (WAKO, Tokyo, Japan) in PBS containing 0.1% Tween-20 (PBS-T) at room temperature for 1 h, and then incubated overnight in goat anti-mouse IL-1ra primary antibody (1∶500; R&D Systems, Inc.) at 4°C. The same concentration of non-immune goat IgG (1∶5000; R&D Systems, Inc.) was used as the negative control. On the following day, the membranes were washed three times (5 min each) with PBS-T, incubated with horseradish peroxidase-conjugated donkey anti-goat IgG (1∶1000; Santa Cruz Biotechnology, CA) for 1 h at room temperature, and washed three times (5 min each) with PBS-T. Protein bands were visualized with the ECL Plus Western blotting detection system (Amersham Pharmacia Biotech, Buckinghamshire, UK).

### Statistical analysis

All data are presented as the mean ± SD. The statistical analyses for the effect of poly I:C or rrIL-1β on spontaneous activity and rectal temperature were performed by two-way analysis of variance (ANOVA). When the differences between groups were significant, comparison of values across groups was further evaluated by one-way ANOVA followed by Scheffe's post-hoc test. Amounts of mRNA in regions of the brain were also submitted to one-way ANOVA and the post-hoc test. *P*<0.05 was considered to be statistically significant.

## Results

**Figure 1:**
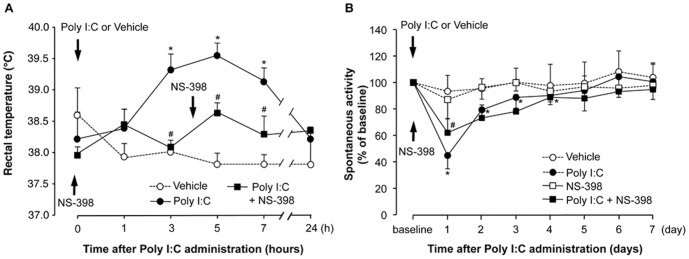
Transient fever and decreased spontaneous activity in response to poly I:C injection, and the effect of NS-398. Rats were injected with the poly I:C (3 mg/kg, i.p.) or the vehicle (saline) at time (*t*)  =  0 min. NS-398 was injected i.p. 5 min before and 4 h after poly I:C injection. (A) The line plot depicts the rectal temperature just before and 1, 3, 5, 7, and 24 h after poly I:C injection. (B) The line plots represent the percent change in spontaneous activity from baseline. Spontaneous activity was measured from the onset of and throughout the dark period. Arrows depict the time of injections. N = 5–8 per group. *P<0.05 versus vehicle-injected group, ^#^P<0.05 versus poly I:C-injected group.

**Figure 2:**
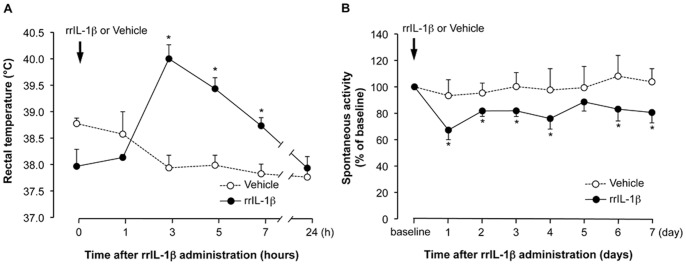
Transient fever and decreased spontaneous activity in response to rat recombinant (rr) IL-1β injection. Rats were injected with rrIL-1β (30 µg/kg, i.p.) or the vehicle (saline) at time (*t*)  = 0 min. (A) The line plots depict the rectal temperature just before and 1, 3, 5, 7, and 24 h after the injection. (B) The line plot represents the percent change in spontaneous activity from baseline. Spontaneous activity was measured from the onset of and throughout the dark period. Arrows depicts the time of injection. N = 5–8 per group. *P<0.05 versus vehicle-injected group.

**Figure 3:**
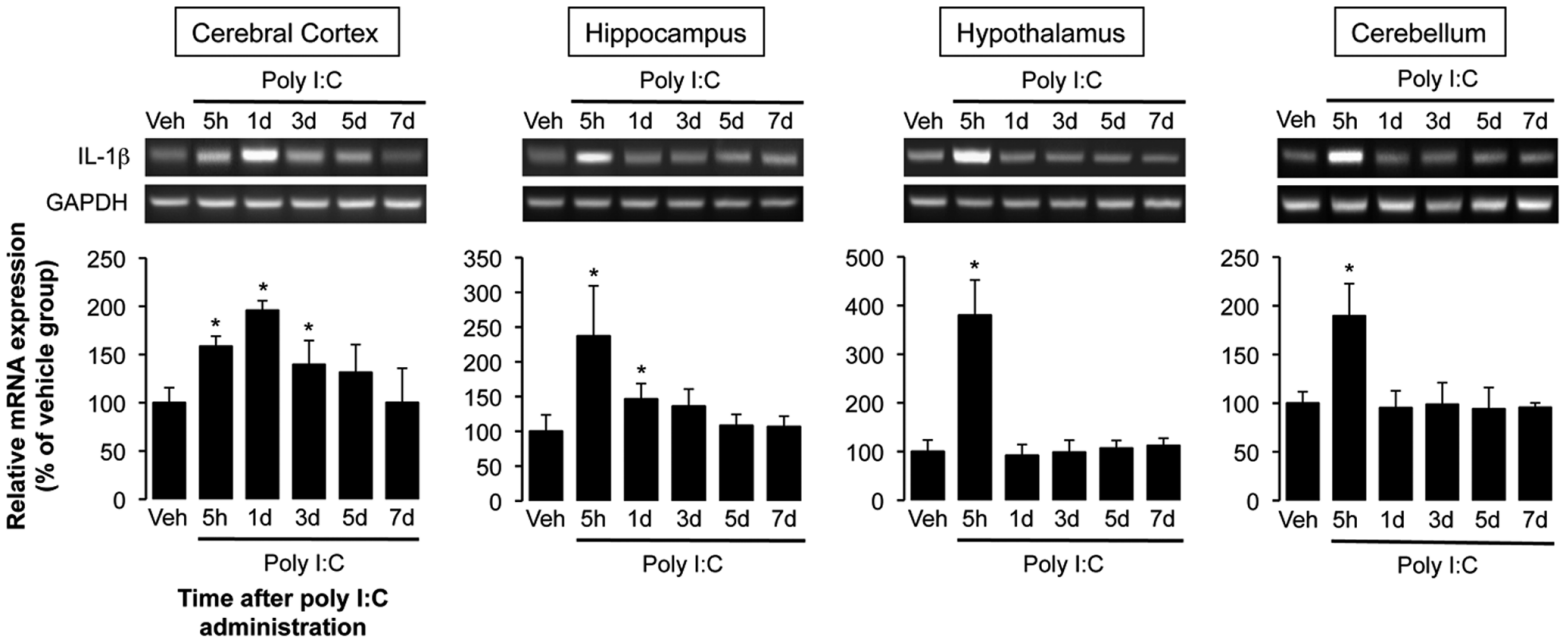
Alteration in IL-1β mRNA expression in the several brain regions following poly I:C injection. Tissue samples (cerebral cortex, hippocampus, hypothalamus, and cerebellum) were prepared 5 h and 1, 3, 5, and 7 days after poly I:C injection. The bar plots show the levels of IL-1β mRNA as the fold change relative to the corresponding vehicle-injected group after calibration with the GAPDH mRNA level. N = 4–5 per experimental group. *P<0.05 versus vehicle-injected group.

Intraperitoneal injection of 3 mg/kg poly I:C induced a significant increase in rectal temperature, which peaked 5 h after poly I:C injection (39.4°C±0.2°C) and then gradually decreased to the normal level within 24 h (Figure 1A). Intraperitoneal injection of NS-398, a COX-2 inhibitor (4 mg/kg twice, 5 min before and 4 h after poly I:C injection), significantly attenuated the fever induced by poly I:C compared to the poly I:C group (Figure 1A; 3, 5, and 7 h after poly I:C injection, P<0.05). Spontaneous activity was also affected by poly I:C injection. On day 1, the spontaneous activity was significantly decreased to 42.2% of baseline (P<0.05 compared to vehicle-injected group), gradually recovered during subsequent days (the decrease was significant compared to the vehicle-injected group until day 4, P<0.05), and almost recovered to the baseline level on day 7 (Figure 1B). We focused on investigating whether poly I:C-induced transient fever affect the spontaneous activity. Injection of NS-398 showed a significant, but partial attenuation of the poly I:C-induced suppression of spontaneous activity only on day 1 (poly I:C + saline group, 42.4%±7.5% baseline; poly I:C + NS-398 group, 62.3%±7.3% baseline; P<0.05), and did not show any attenuation on subsequent experimental days. Injection of NS-398 into animals not given poly I:C did not affect spontaneous activity (Figure 1B). Intraperitoneally injected poly I:C is recognized mainly by intracellular TLR3, which is expressed in the periphery, and pro-inflammatory cytokines are then produced and released into the circulation. We investigated whether IL-1β, one of the major inflammatory cytokines, induces fever and decreased spontaneous activity, and we found that intraperitoneal injection of rrIL-1β (30 µg/kg) induced fever and decreased the spontaneous activity (Figure 2). The rectal temperature reached the peak earlier (3 h after rrIL-1β injection) and was higher (40.0°C±0.3°C) than that induced by poly I:C injection (Figure 2A). Spontaneous activity was also significantly decreased by rrIL-1β injection compared to the vehicle-injected group on days 1, 3, 5, and 7 after injection (P<0.05). The magnitude of the decrease was lower than that in the poly I:C-injected group on day 1 (67.3%±7.3% baseline), but the spontaneous activity had not recovered within 7 days (Figure 2B).

To examine the brain mechanisms underlying the poly I:C-induced decrease in spontaneous activity, tissues from various brain regions (cerebral cortex, hippocampus, hypothalamus, and cerebellum) of saline-injected and poly I:C-treated rats were obtained, and the expression of IL-1β mRNA was analyzed with RT- PCR in each tissue (Figure 3). At 5 h after poly I:C injection, IL-1β mRNA expression was significantly up-regulated in each region of the brain examined (158.4%, cerebral cortex; 237.1%, hippocampus; 380.0%, hypothalamus; 189.5%, cerebellum; compared with control group, P<0.05). On day 1 (24 h after poly I:C injection), IL-1β mRNA expression in the cerebral cortex showed the highest level (195.8%, P<0.05 compared to control group) and gradually decreased, although the level was higher than the control level in saline-injected rats even 3-5 days after poly I:C injection. The expression in regions other than the cerebral cortex recovered to control levels within 1–3 days after poly I:C injection. The expression of IL-1β mRNA in the brain was up-regulated by i.p. injection of rrIL-1β similarly to poly I:C injection (Figure S1), and the overexpression of IL-1β mRNA lasted longer than that by poly I:C injection in cerebral cortex.

To investigate if IL-1β production in the brain is involved in decreased spontaneous activity, we performed i.c.v. infusion of rrIL-1β (30 ng/day). The dose of rrIL-1β was defined based on a previous study by Cao et al. [19]. Infusion of rrIL-1β dramatically decreased spontaneous activity (Figure S2). After the end of infusion for 24 h, the decreased activity was not recovered and animals were very exhausted with severe weight loss. In this experiment, some animals underwent euthanized by deep anesthesia, according to the ethical rule (Figure S2A). Thus, we investigated the effect of a lower dose of rrIL-1β on spontaneous activity. As shown in Figure S2B, i.c.v. infusion of rrIL-1β only for 16 h decreased the spontaneous activity on day 1. The activity was recovered within 5 days.

**Figure 4:**
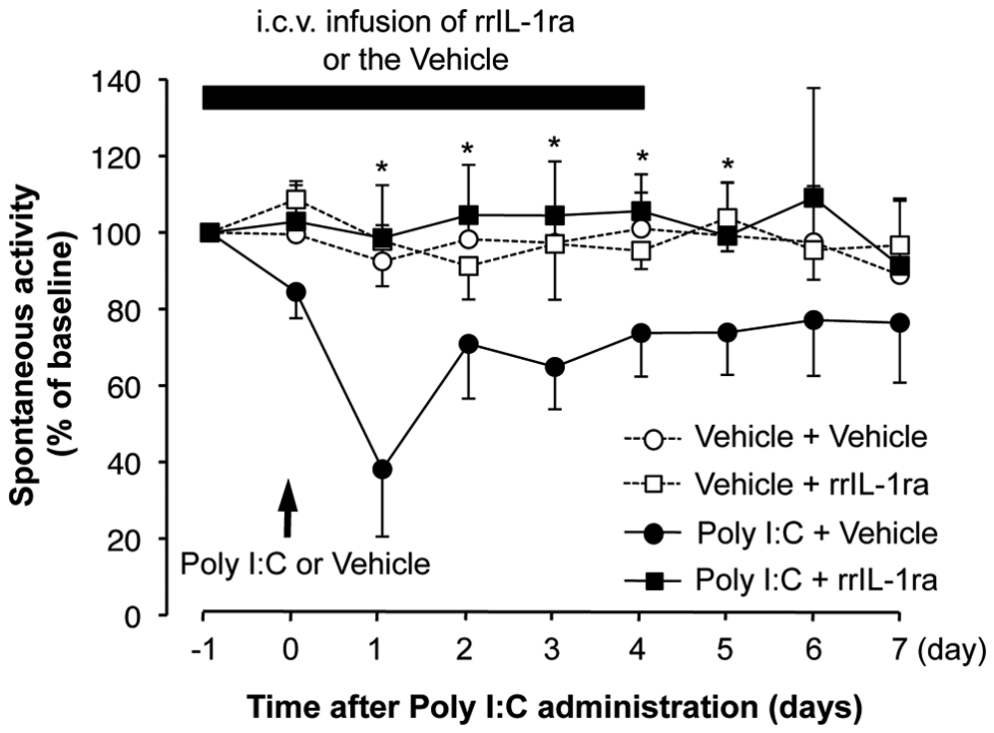
Effect of i.c.v. infusion of rrIL-1ra on poly I:C-induced decrease in spontaneous activity. Poly I:C-induced spontaneous activity in the presence of i.c.v. infusion of rat recombinant IL-1 receptor antagonist (rrIL-1ra, 1 µg/day). Infusion (at a rate of 1 µl/h) was started 1 day before poly I:C injection and continued for 5 days. The line plots represent the percent change in spontaneous activity from baseline. Spontaneous activity was measured from the onset of and throughout the dark period. Arrows depict the time of poly I:C injection. N = 5–8 per experimental group. *P<0.05, versus poly I:C + vehicle infusion group.

Next, to determine whether the poly I:C-induced decrease in spontaneous activity was induced by intrinsic IL-1β in the brain, rats were injected with poly I:C during i.c.v. infusion of rrIL-1ra (receptor antagonist; 1 µg/day for 5 days) or vehicle (0.1% serum albumin in 0.1 M PBS) (Figure 4). The i.c.v. infusion was automatically started by the subcutaneously implanted minipump 24 h before poly I:C injection and was maintained for 5 days. The dose of rrIL-1ra that was i.c.v. infused was defined based on a previous study by Koo and Duman [20]. Intracerebroventricular infusion of rrIL-1ra completely attenuated the poly I:C-induced decrease in spontaneous activity. Intracerebroventricular infusion of rrIL-1ra into rats not given poly I:C did not show any change in spontaneous activity (Figure 4).

**Figure 5:**
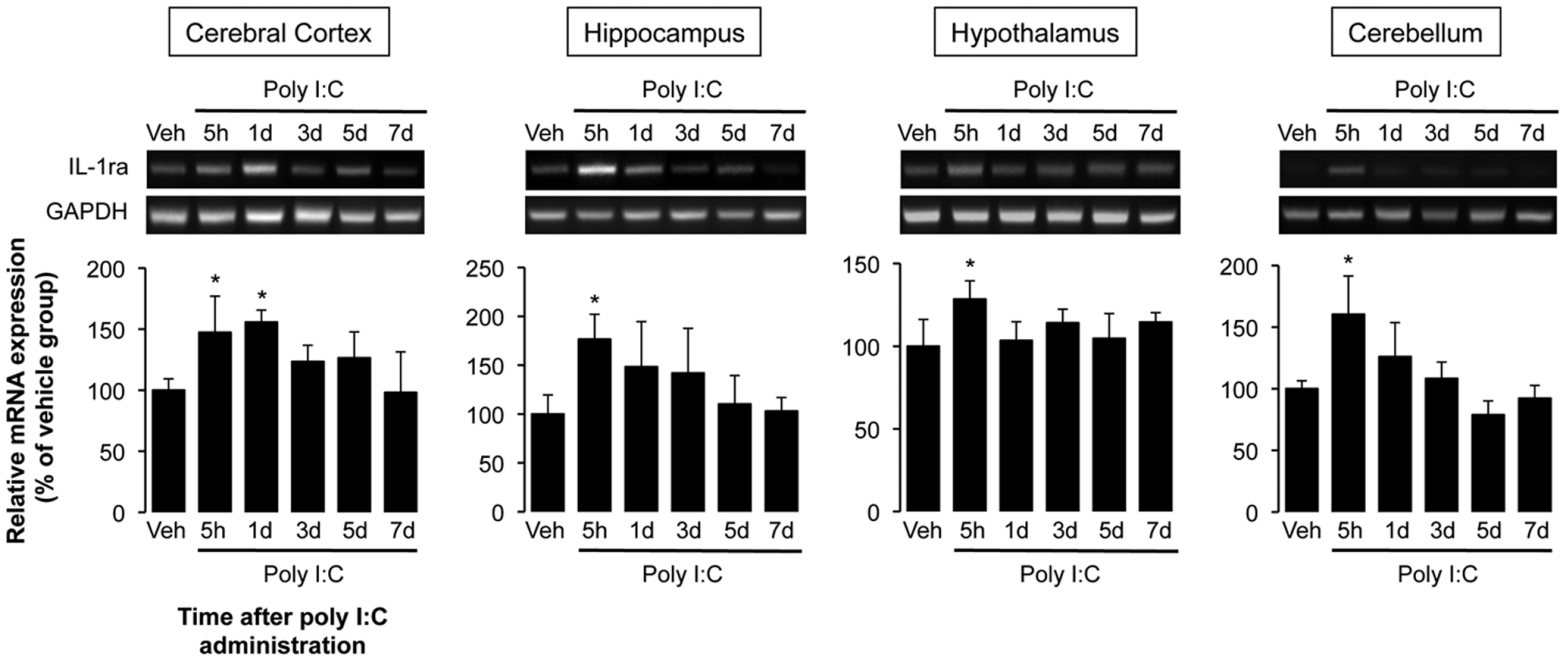
Effect of poly I:C on IL-1ra mRNA expression in the various brain regions. Tissue samples (cerebral cortex, hippocampus, hypothalamus, and cerebellum) were prepared 5 h and 1, 3, 5, and 7 days after poly I:C injection. The bar plots show the levels of IL-1ra mRNA as the fold change relative to the corresponding vehicle-injected group after calibration with the GAPDH mRNA level. N = 4–5 per experimental group. *P<0.05 versus vehicle-injected group.

IL-1ra is an endogenous competitive antagonist for IL-1 receptors and counteracts the pro-inflammatory responses of IL-1β [21]. To investigate the role of intrinsic IL-1ra in the poly I:C-induced decrease in spontaneous activity, we first confirmed the expression of IL-1ra mRNA in various brain regions (cerebral cortex, hippocampus, hypothalamus, and cerebellum) of poly I:C-injected animals. As shown in Figure 5, the expression of IL-1ra mRNA was significantly increased in those brain regions 5 h after injection of poly I:C (cerebral cortex, hippocampus, hypothalamus, and cerebellum, P<0.05 compared to control group). On day 1, the expression of IL-1ra was significantly higher than that in control animals in the cerebral cortex (P<0.05), and tended to increase until day 5.

**Figure 6:**
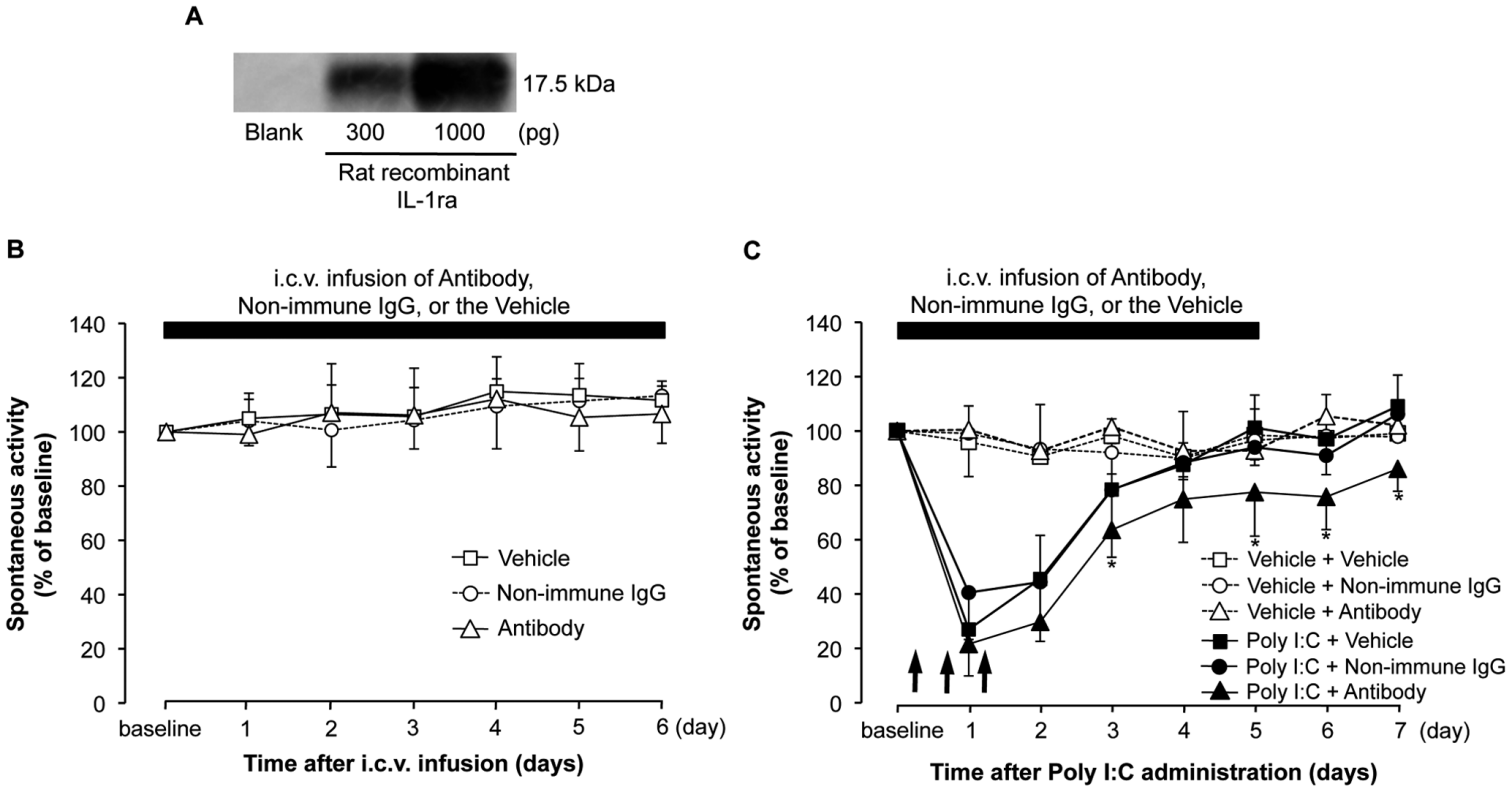
Effect of i.c.v. infusion of anti-IL-1ra neutralizing antibody on poly I:C-induced decreased spontaneous activity. (A) Immunoblotting result. rrIL-1ra (300, 1000 pg) was detected by cross-reactivity of the anti-mouse neutralizing antibody. Infusion (100 ng/µl, at a rate of 1 µl/h) was started 7 days before poly I:C injection and continued for 5 days. (B) Before injection and (C) after injection of poly I:C or the vehicle. The line plots represent the percent change in spontaneous activity from baseline. Spontaneous activity was measured from the onset of and throughout the dark period. N = 4–8 per experimental group. Arrows depict the time of poly I:C or the vehicle injection. *P<0.05 versus poly I:C + i.c.v. infusion of non-immune IgG group. In this procedure, we injected poly I:C or the vehicle three times (10:00, 18:00, and 10:00 on the next day) to precisely evaluate the effect of neutralizing antibody on the recovery speed of spontaneous activity. We infused antibody from 7 days before poly I:C injection, due to slow diffusion speed of antibody with a big chemical structure.

To observe the role of intrinsically produced IL-1ra in the brain on the poly I:C-induced decrease in spontaneous activity, we suppressed the action of intrinsic IL-1ra with a neutralizing antibody for IL-1ra, which was an anti-mouse IL-1ra antibody. According to the manufacturer's product information, the antibody shows potent cross-reactivity against rrIL-1ra. We confirmed that this antibody recognized rrIL-1ra with immunoblotting. Figure 6 shows the effect of i.c.v. infusion of the neutralizing antibody or non-immune IgG on the poly I:C-induced decrease in spontaneous activity. Intracerebroventricular infusion of the antibody or non-immune IgG was started 7 days before poly I:C injection and continued 5 days after injection. As shown in Figure 6C, recovery from the poly I:C-induced decrease in spontaneous activity was significantly delayed by i.c.v. infusion of the neutralizing antibody (P<0.05, compared to poly I:C + i.c.v. infusion of non-immune IgG). Infusion of the neutralizing antibody, non-immune IgG, or the vehicle did not produce any significant changes in spontaneous activity in rats not injected with poly I:C (Figure 6B).

**Figure 7:**
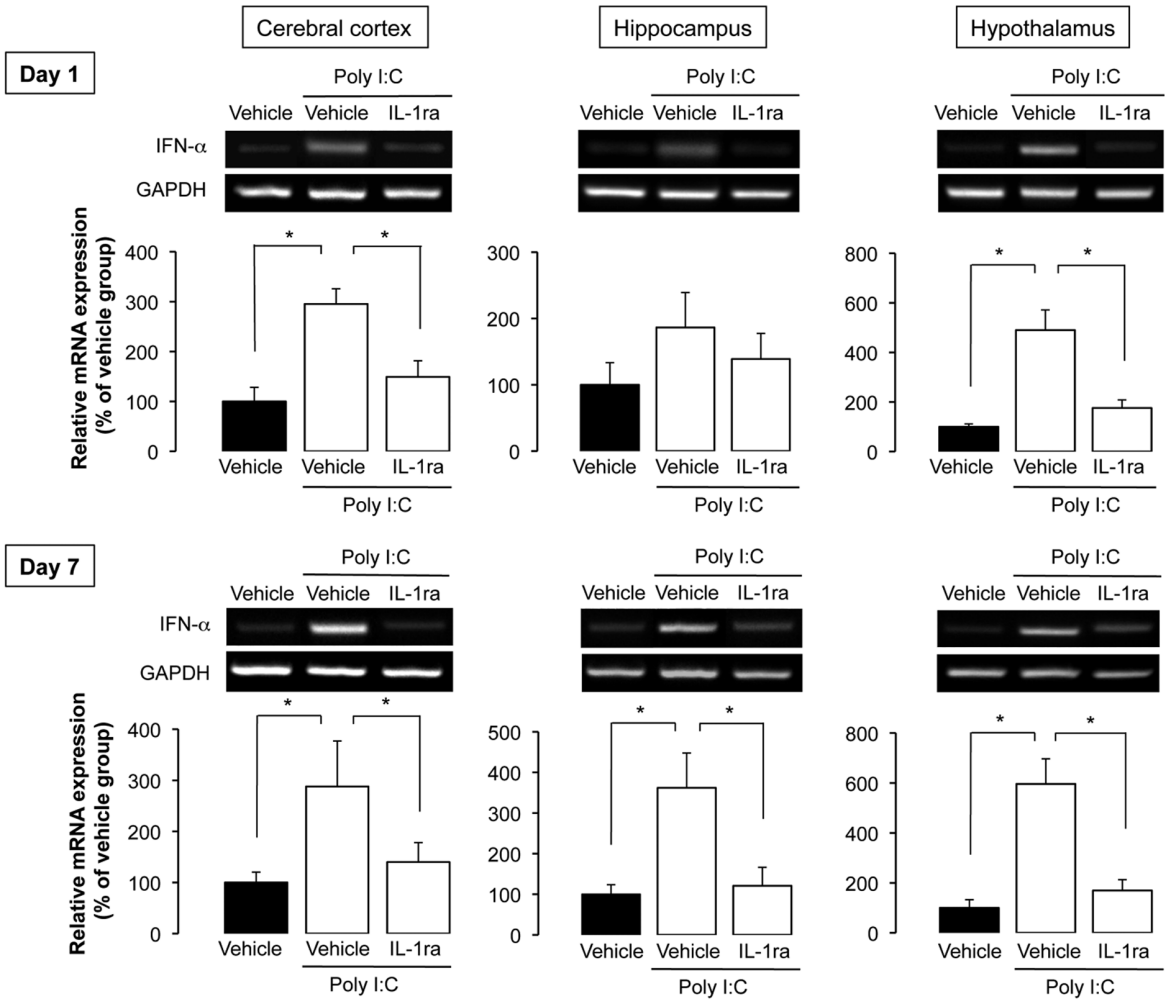
Effect of i.c.v. infusion of rrIL-1ra on brain IFN-α mRNA expression in the brain. On day 1 and day 7 after poly I:C injection, tissue samples (cerebral cortex, hippocampus, and hypothalamus) were prepared. The bar plots show the levels of IFN-α mRNA as the fold change relative to the corresponding vehicle-injected group after calibration with the GAPDH mRNA level. N = 4–5 per experimental group. *P<0.05.

Katafuchi et al. [7] reported that IFN-α expression in the brain plays a key role in poly I:C-induced suppression of spontaneous activity. We investigated whether poly I:C-induced IFN-α expression in the brain was mediated by IL-1β. Poly I:C-induced IFN-α mRNA expression in various regions of the brain (cerebral cortex, hippocampus, and hypothalamus) was significantly attenuated by i.c.v. infusion of rrIL-1ra on day 1 (Figure 7). IFN-α mRNA overexpression was observed in the brain even 7 days after poly I:C injection, which is consistent with a previous report by Katafuchi et al. [7]. However, i.c.v. infusion of rrIL-1ra significantly blocked IFN-α mRNA expression on day 7 (Figure 7). Intraperitoneal injection of rrIL-1β also induced overexpression of IFN-α mRNA in the brain (Figure S1).

## Discussion

The present results show that activation of the IL-1 receptor in the brain is the primary mechanism of action for the poly I:C-induced decrease in spontaneous activity. Moreover, IL-1ra, an endogenous competitive inhibitor of the IL-1 receptor, plays an important role in relief of neuroinflammation involved in IL-1β-induced depressive symptoms.

In the present study, suppression of spontaneous activity was measured in rats by evaluating decreased spontaneous activity. Spontaneous activity is reported to be an acceptable surrogate marker of fatigue or fatigability in the rat [22]. Katafuchi et al. [7] has evaluated “immunologically induced fatigue” by measuring running wheel activity, which is rather sensitive to motivation. In the present study, each rat was isolated and habituated in a home cage, and spontaneous activity was measured for a week, minimizing the effect of extrinsic motivation. Therefore, decreased spontaneous activity in our study can be interpreted as a measure of “fatigue-sensation”. Poly I:C-injected animals were reported to show a prolonged decrease in running wheel activity for more than a week [7]. Our results demonstrated that decreased spontaneous activity peaked on day 1 and gradually recovered during the following days. Running wheel activity is thought to be more potently affected by motivation than spontaneous activity. These results suggest that motivation requires a longer time for recovery.

Fortier et al. [23] reported that poly I:C-induced transient fever depends on COX-2 overexpression in the hypothalamic area. In the present study, we also observed transient fever, which peaked 5 h after poly I:C injection and completely declined within 24 h after injection. The fever was arrested by i.p. injection of NS-398, a COX-2 inhibitor, corresponding to a previous study by Fortier et al. In our study, the dose (4 mg/kg) and preparation of NS-398 were determined based on a previous report [19]. We administered two doses of NS-398 5 min before and 4 h after poly I:C injection, because of the short half-life of efficacy of NS-398. The poly I:C-induced decrease in spontaneous activity was partially (about 20%) relieved by NS-398 injection only on the first day after poly I:C injection, whereas NS-398 itself did not affect physiological spontaneous activity. These data suggest that transient fever has a certain potency for suppressing spontaneous activity. Porkka-Heiskanen et al.[24] demonstrated that adenosine is a sleep-inducing mediator, and that brain adenosine levels are elevated at a time when circadian body temperatures are high. Furthermore, extracellular adenosine is well known to act on presynaptic adenosine A1 receptors to inhibit excitatory synaptic transmission [25], [26]. These reports suggested that the temperature-dependent increase in extracellular adenosine induces such a suppressive effect on spontaneous activity.

Peripheral immune stimulation acts on the brain and induces sickness behavior [10]. We observed that i.p. injection of rrIL-1β produced transient fever and decreased spontaneous activity similary to poly I:C injection. These results suggested that peripheral immune challenge or peripheral inflammation leads to cytokine induction in the brain. Poly I:C injection induces IL-1β upregulation mainly in the hypothalamic area as an acute response [9], [23]. In the present study, IL-1β mRNA in the hypothalamus peaked 5 h and decreased 24 h after poly I:C injection, which paralleled the transient fever. Therefore, these results indicated that overexpression of hypothalamic IL-1β mRNA may be involved in transient fever. Also, in the cerebral cortex and hippocampus, IL-1β mRNA expression was significantly increased compared to vehicle-injected group on day 1 after poly I:C injection. On days 3 and 5, the expression level shows trend higher than that in vehicle-injected group.

Recent studies including neuroimaging have revealed that the cortical region receives information about inflammatory processes in the body [27], and cortical function is modulated by systemic inflammation [28], [29]. Also, using positron emission tomography, Hannestad et al. [30] demonstrated that glucose metabolism in the cerebral cortex, particularly in the insular and cingulate cortex, is affected by systemic endotoxin treatment. Katafuchi et al [7]. have reported that peripheral injection of poly I:C suppresses voluntary running wheel activity by prolonged upregulation of IFN-α mRNA in the cerebral cortex, hippocampus, and hypothalamic regions. These reports indicate that peripheral inflammation induces neuroinflammation in the brain, including the cerebral cortex, and produces fatigue-like behavior.

In the present study, i.c.v. infusion was carefully and precisely performed using the programmable minipump “iPRECIO” (see Materials and Methods). The present results showed that i.c.v. infusion of rrIL-1ra (1 µg/day for 5 days) completely attenuated the poly I:C-induced decrease in spontaneous activity. IL-1 and IL-1ra rapidly reach the circumventricular organs, hypothalamus, and amygdala after i.c.v. administration, but only approximately 1% of the original amount is still present in the brain 2 h later [31]. Furthermore, Konsman et al. [32] demonstrated that 0.05 to 0.15% of i.c.v.-administered IL-1ra can be detected in the plasma 1.5 and 3.5 h later. In fact, i.c.v.-administered IL-1β is also rapidly cleared from the brain, resulting in increased plasma and liver levels [31], [33]. These reports suggest that i.c.v.-administered rrIL-1ra may affect the periphery via the circulation. Fortier et al. [23] reported that i.p. injection of recombinant human IL-1ra at a dose more than 1.0 mg/kg is required to block fever induced by poly I:C injection. If IL-1ra injected at a dose of 1.0 mg/kg uniformly diffuses in the body of a rat with a body weight of 260 g which is comparable to a body mass of 260 ml, the final concentration can be calculated as 1.0 µg/ml. Next, Circulated blood volume was thought as approximately 1/13 of body mass in animals. Therefore, the circulating blood volume of 260 g rat is caluculated to 20 ml. If almost all i.c.v.-infused rrIL-1ra (5 µg) flows into the circulation, the concentration of rrIL-1ra in the circulation can be evaluated as 0.25 µg/ml, which is less than the pharmacologicaly effective concentration (1.0 µg/ml, approximately 1.0 mg/kg in the report by Fortier et al. [23]). These estimations indicate that i.c.v.-infused IL-1ra was likely effective only in the brain.

Poly I:C is a potent inducer of IFN-α in vivo and in vitro [34], [35]. Morikawa et al. [8] demonstrated that IFN-α treatment increased the expression of 5-HTT mRNA in cultured cells. Katafuchi et al. [9] reported that production of IFN-α in the brain is involved in poly I:C-induced fatigue-like behavior due to overexpression of 5-HTT, which suppresses serotonergic neurotransmission. In that study, poly I:C was injected into the periphery, and it is unclear if IFN-α expression in the brain was induced by poly I:C directly or via peripheral inflammation. In the present study, i.c.v. infusion of rrIL-1ra prior to poly I:C injection significantly blocked IFN-α overexpression in the brain on days 1 and 7. These results suggest that IFN-α overexpression in the brain is triggered by poly I:C-induced IL-1β expression in the brain. We also showed that i.p. injection of rrIL-1β decreased spontaneous activity for a week, accompanied by expressions of IL-1β and IFN-α in the brain.

Fatigue sensation often occurs in patients receiving IFN-α for treatment for hepatitis C. Loftis et al. [36] reported that the depressive symptoms correlate with elevated plasma levels of IL-1β and TNF-α in patients with chronic hepatitis C. Also, Kaneko et al. [37] reported that IL-1β-immunopositive cells are increased in the dentate gyrus in animals receiving subchronic i.p. injection of IFN-α. Therefore, peripheral IFN-α treatment-induced depressive symptoms including fatigue sensation may involve IL-1β in the brain.

The IL-1ra is a member of the IL-1 family that binds to IL-1 receptors, but does not induce an intracellular response. Some reports have been published on middle cerebral artery occlusion (MCAO)-induced brain lesions. Loddick et al. [15] demonstrated that i.c.v. injection of anti-IL-1ra antiserum aggravates brain lesions induced by MCAO in rats, indicating that endogenous IL-1ra shows a neuroprotective effect in the brain. In the present study, IL-1ra mRNA was overexpressed in the brain following poly I:C injection, and the expression pattern paralleled that of IL-1β, especially in the cerebral cortex. We also demonstrated that i.c.v. infusion of anti-IL-1ra antibody significantly delayed the recovery from decreased spontaneous activity induced by poly I:C injection. These results indicate that endogenous IL-1ra in the brain prevents prolonged inflammation. Recent studies have revealed that neuroinflammation is an important precipitating event in various neurological disorders including Alzheimer's disease and depression [38], [39]. Therefore, a balance in IL-1β and IL-1ra productivity in the brain is intimately involved in the pathogenesis of neurological diseases. Indeed, a recent report suggests that IL-1ra may be a new candidate for treating depression [40]. The present report increases our understanding of how neuroinflammation could shift from acute to chronic state following transient virus infection.

## Supporting Information

Figure S1 **Effects of i.p. injection of rat recombinant (rr) IL-1β on brain IL-1β and IFN-α mRNAs expressions.** Tissue samples (cerebral cortex, hippocampus, hypothalamus, and cerebellum) were prepared 3 h, 1 d, 3 d, and 7 d after rrIL-1β injection. The bar plots show the levels of IL-1β and IFN-α as the fold change relative to the corresponding vehicle-injected group after calibration with the GAPDH mRNA level. N = 3–5 per experimental group. *P<0.05 versus vehicle-injected group.
(TIF)

Figure S2 **Effect of i.c.v. infusion of rrIL-1β on spontaneous activity.** The line plots represent the percent change in spontaneous activity from baseline. Spontaneous activity was measured from the onset of and throughout the dark period. 30 ng/day for 24 h (A) and for 16 h (B) at the rate of 1 µl/h. N = 3–4 per experimental group. *P<0.05 versus vehicle infusion group. Arrowheads in (A) indicate euthanization.
(TIF)
