# Supplementary figures and images for: Brain Interleukin-1β and the Intrinsic Receptor Antagonist Control Peripheral Toll-Like Receptor 3-Mediated Suppression of Spontaneous Activity in Rats
Source: PLoS One. 2014 Mar 12;9(3):e90950. doi: 10.1371/journal.pone.0090950 (PMC3951245; doi:10.1371/journal.pone.0090950)

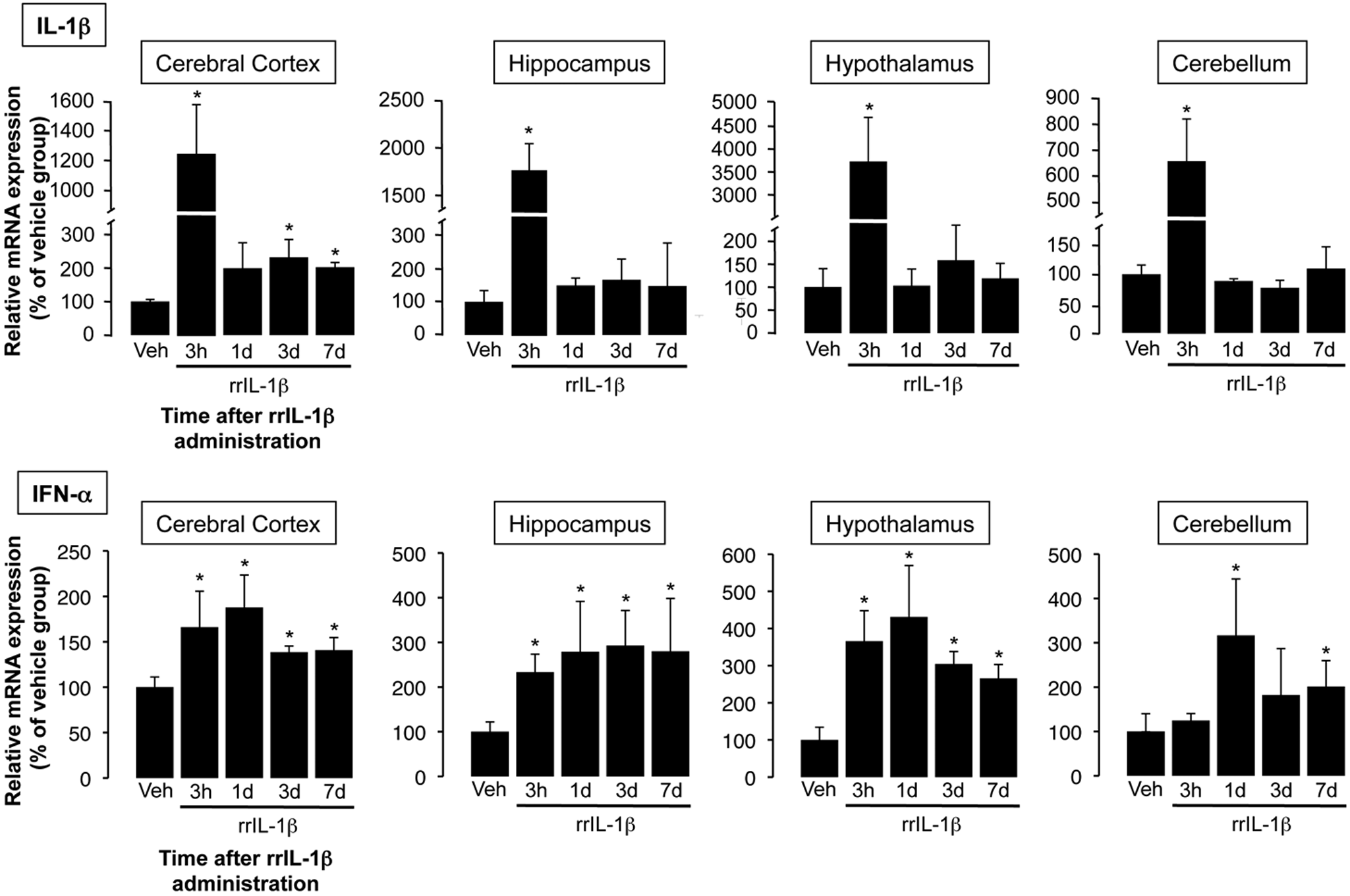

Supplement: Figure S1 — Effects of i.p. injection of rat recombinant (rr) IL-1β on brain IL-1β and IFN-α mRNAs expressions. Tissue samples (cerebral cortex, hippocampus, hypothalamus, and cerebellum) were prepared 3 h, 1 d, 3 d, and 7 d after rrIL-1β injection. The bar plots show the levels of IL-1β and IFN-α as the fold change relative to the corresponding vehicle-injected group after calibration with the GAPDH mRNA level. N = 3–5 per experimental group. *P<0.05 versus vehicle-injected group. (TIF) [file pone.0090950.s001.tif]

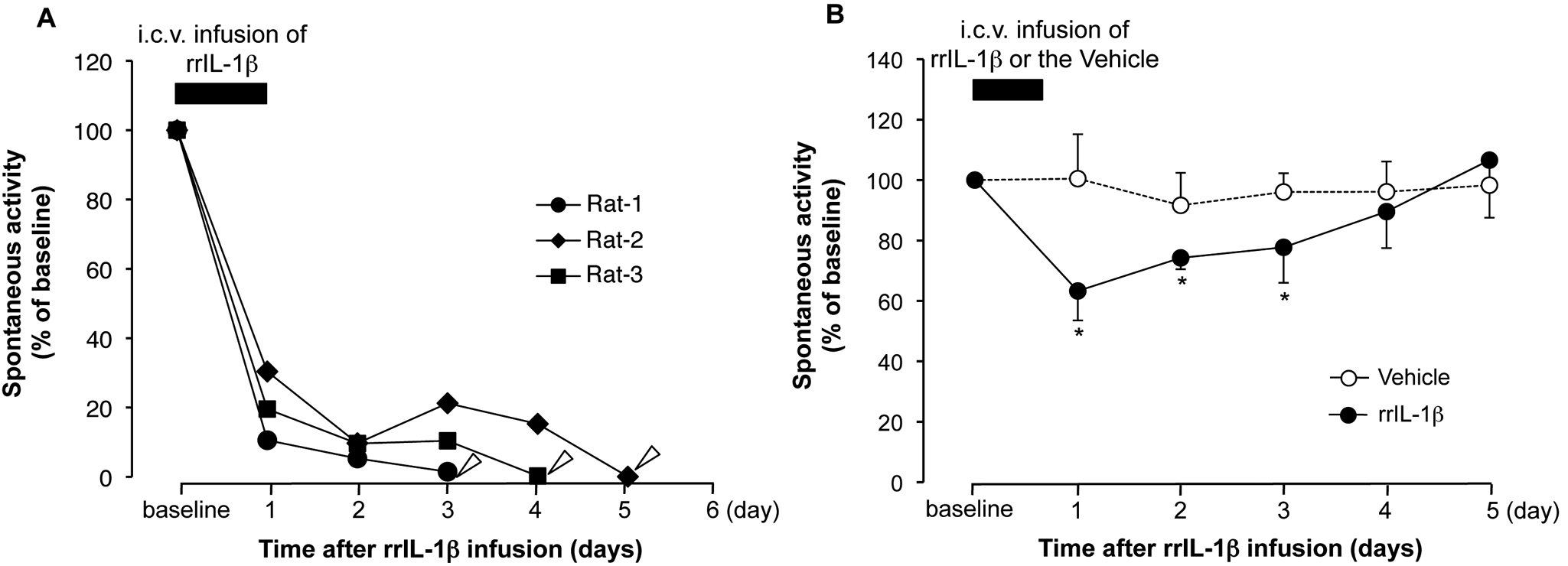

Supplement: Figure S2 — Effect of i.c.v. infusion of rrIL-1β on spontaneous activity. The line plots represent the percent change in spontaneous activity from baseline. Spontaneous activity was measured from the onset of and throughout the dark period. 30 ng/day for 24 h (A) and for 16 h (B) at the rate of 1 µl/h. N = 3–4 per experimental group. *P<0.05 versus vehicle infusion group. Arrowheads in (A) indicate euthanization. (TIF) [file pone.0090950.s002.tif]
